# Dr. Simons, on the Distillation of Vinegar

**Published:** 1800-11

**Authors:** B. B. Simons

**Affiliations:** Edinburgh


### *To the Editors of the Medical and Physical Journal.*

Gentlemen,

SHOULD you think the following mode of treating the residuum of vinegar, in the process Acetum Distillatum of the Edinb. College, merits a place in the Journal, please to insert it.

*Edinburgh, Aug.* 13, 1800. B. B. SIMONS, M. D.

---

The College orders "eight pounds of vinegar to be distilled in glass vessels with a gentle heat; let the two first pounds that

---

that come over be thrown away, as containing too much water; let the four pounds next following be reserved as the distilled vinegar. What remains is a still stronger acid, but being too much burnt is unfit for use." Some chemists, rather than throw away the residuum, add either the water that first passes over, containing a small quantity of vinegar, or a portion of fresh, and distil. The vinegar thus obtained is never free from the empyreuma. Instead of adding water, I added about ℥iiij of finely powdered charcoal, previously heated and distilled to dryness, raising the heat of the furnace as the process advanced. The residuum, thus treated, was entirely deprived of empyreuma, and was thought by a gentleman, who tasted of several quantities taken from different periods of the process, to be superior in flavour. It is much concentrated, and, when added to the four pounds, is better calculated for pharmaceutical purposes. The price of charcoal may seem to militate against this mode of treating the residuum; but it cannot, for if the coal be washed, and dried in a tolerably strong heat, the same effect will take place when added to the residuum of another process. I have not ascertained the precise quantity of coal necessary to correct the empyreuma, but, I presume, a much smaller may be employed.

